# Drugs against broad-spectrum of coronaviruses

**DOI:** 10.3389/fimmu.2026.1728474

**Published:** 2026-04-13

**Authors:** Shaoli Huang, Xihe Zhang, Wenying Luo, YueHao Yao, Juan Hu, Xaoyan Wang, Hongwu Xin

**Affiliations:** 1 Clinical Laboratory, People’s Hospital of Lianjiang, Lianjiang, Guangdong, China; 2 Clinical Laboratory Area 2, Affiliated Hospital of Guangdong Medical University, Zhanjiang, Guangdong, China; 3 Doctoral Scientific Research Center, People's Hospital of Lianjiang, Lianjiang, Guangdong, China; 4 The Doctoral Scientific Research Center, People’s Hospital of Lianjiang, Guangdong Medical University, Lianjiang, Guangdong, China; 5 Department of Respiratory Medicine, Clinical Laboratory, People’s Hospital of Lianjiang, Lianjiang, Guangdong, China; 6 Key Laboratory of Biomedical Engineering Technology at Universities of Shandong Province, Special Laboratory of Medical Biotechnology and Functional Materials of Shandong Province, and Department of Pathology and Pathophysiology, College of Basic Medical Sciences, Qilu Medical University, Zibo, Shandong, China; 7 Key Laboratory of Research on Human Genetic Diseases Research at Universities of Inner Mongolia Autonomous Region, School of Basic Medical Sciences, Chifeng University, Chifeng, Inner Mongolian Autonomous Region, China

**Keywords:** broad-spectrum antiviral drug, coronavirus, macromolecule, small molecule, traditional Chinese medicine

## Abstract

The continuous emergence of severe acute respiratory type 2 coronavirus (SARS-CoV-2) variants (e.g., Omicron) and the threat of future emerging coronavirus pandemics highlight the urgent need for broad-spectrum antiviral strategies. While various therapeutics exist, a systematic integration of diverse treatment modalities remains lacking. This review introduces a comprehensive conceptual framework that compares and integrates four major therapeutic categories: small-molecule drugs (targeting viral enzymes), macromolecular drugs (including peptides and polymers), Traditional Chinese Medicine (TCM, focusing on holistic regulation and active ingredients), and carrier vector vaccines. Beyond traditional pharmacology, we further incorporate the emerging role of Artificial Intelligence (AI) and computational screening in accelerating the discovery of broad-spectrum inhibitors. The primary goal of this article is to: (1) critically analyze the distinct antiviral mechanisms, advantages, and limitations of each category; (2) explore synergistic combination therapies (e.g., combining antiviral drugs with immunomodulators or TCM) to overcome drug resistance; and (3) provide a strategic reference for developing “pan-coronavirus” therapeutics that are resilient against viral mutations.

## 1 Introduction

The severe acute respiratory type 2 coronavirus (SARS-CoV-2) infection has caused the Coronavirus Disease 2019 (COVID-19) pandemic, which has grown into an unparalleled worldwide health emergency. It has caused 531 million total infections and 6.3 million deaths globally, according to WHO estimate revised June 9, 2022 (1). The clinical spectrum of COVID-19 ranged from severe pneumonia with respiratory failure to asymptomatic infection. The degree of cytokine storm is substantially associated with lung injury, multiple organ failure, and poor prognosis (2, 3). By activating epithelial, endothelial, and alveolar macrophages through pattern recognition receptors, COVID-19 release damage-associated molecular patterns (DAMPs), which sets off proinflammarory cascades that draw in innate and adaptive immune cells via chemokine gradients. The cytokines and chemokines secreted by recruited cells create positive feedback loops that exacerbate tissue injury and inflammation (4, 5).

Despite the widespread distribution of COVID-19 vaccines, it remains challenging due to emerging SARS-CoV-2 variants like Alpha, Beta, Gamma, Delta, and Omicron. These variants often have mutations in the spike protein, the main target of vaccines, which can reduce vaccine efficacy. SARS-CoV-2 drugs include small-molecule drugs, macromolecular drugs and traditional Chinese medicine. Nanocarriers, such as virus-like particles, are being studied for vaccine and drug delivery.

Public health were significantly impacted by the SARS, MERS and COVID-19 pandemics. The development of broad-spectrum antiviral drugs against coronaviruses is one of the primary efforts to combat the future pandemic of diseases caused by existing and emerging coronaviruses. As of now, the search for clinically approved broad-spectrum antiviral drugs for β-coronaviruses, including SARS-CoV-2, continues. This unmet medical need has spurred a flurry of research activity around the world (6).

## 2 Coronavirus structures and drug design strategies

Coronaviruses (CoVs) are single-stranded, positive-strand RNA viruses with membrane spikes and range in size from 60 to 140 nm. Their RNA genomes are roughly 29.8 kb and contain 14 open reading frames (ORFs) that encode 29 proteins, including 9 helper proteins (3a, 3b, 6, 7a, 7b, 8, 9b, 9c, and 10), four structural proteins (spike or S, envelope or E, membrane or M, nucleocapsid or N), and sixteen non-structural proteins (NSP1-16) (7). Coronaviruses are members of the Coronaviridae family and may infect a variety of hosts, causing illnesses that range from common colds to life-threatening diseases (8).

Coronaviruses fuse with the host cell membrane through their spike (S) proteins, releasing their genome for replication. The spike protein consists of two subunits, S1 and S2, and the S1 subunit interacts with the cellular receptor, while the S2 subunit promotes membrane fusion. Most coronaviruses use the C-terminal domain of the S1 subunit as the receptor-binding domain (RBD) (9). The viral S protein is the main target of currently available vaccines and drugs, including antibody therapies (10–13). Unfortunately, the newly discovered SARS-CoV-2 variations are mostly located in the S protein (14).

Angiotensin-converting enzyme 2 (ACE 2) is the cellular receptor for SARS-CoV-2 and some other coronaviruses (14, 15). Recent studies have shown that RBD-ACE2 binding is a critical virion entry determinant, enabling early-stage antiviral intervention through receptor blockade (16, 17). The early fusion intermediate conformation (E-FIC) could be an ideal target for the creation of a dual-functional antiviral drug, and the protein AL5E (E-FIC-targeted bifunctional antiviral protein) may be a useful human coronaviruses inhibitor and inactivator (15).

Viral N proteins, which facilitate genomic RNA encapsulation, demonstrate notable evolutionary stability, which is a good target to develop broad spectrum of anti-coronavirus drugs. Recently, the researchers found that the N protein engages with host-derived deaminase enzymes through co-localization within cellular stress granules, which significantly elevates the mutation frequency of the viral RNA transcriptome (18).

RNA-dependent RNA polymerase (RdRp) is responsible for replicating the viral RNA. The sequence and structure of RdRp are highly conserved among different coronaviruses, making it an attractive target for developing drugs against broad spectrum of coronaviruses. Researchers have been studying the structures of SARS-CoV-2 RdRp and its associated exonuclease (ExoN) protein, as well as the N-exonuclease of Lassa virus. Through advanced techniques such as X-ray crystallography and cryo-electron microscopy, they have been able to determine the three-dimensional structures of these proteins with high precision. By comparing these structures, they have deduced a model of the catalytically active SARS-CoV-2 enzyme. This model provides valuable insights into how RdRp functions and how it can be targeted by drugs (19). For example, it has revealed the key amino acid residues involved in the binding of nucleotides during RNA synthesis. Drugs that can specifically bind to these residues and inhibit nucleotide binding or the polymerase activity can potentially block viral RNA replication.

Coronaviruses are known for its high mutation rate. These mutations can occur in any genes encoding viral proteins, such as the spike protein, N proteins, main protease (Mpro), and RNA-dependent RNA polymerase (RdRp). When mutations occur in the binding sites of existing drugs, the drugs may no longer be able to bind effectively, leading to drug resistance. To develop broad-spectrum small-molecule drugs against coronaviruses, researchers are focusing on developing drugs that target conserved regions of the virus. These conserved regions are less likely to mutate, as they are essential for the virus’s life cycle (20).

## 3 Small-molecule drugs against broad-spectrum of coronaviruses

### 3.1 Overview

Chemical libraries, both natural and synthetic, are in search of novel compounds with potential anti-coronavirus activity. Computational methods, such as virtual screening, are also being used to predict which molecules are most likely to interact with key viral proteins. Optimized orthogonal cell-free assay allows to screen large numbers of small molecules in a cell-free environment, which is more efficient and cost-effective than traditional cell-based assays. Using this method, researchers have identified several small molecules that can inhibit the binding sites of SARS-CoV-2-Mpro and HCoV-OC43-Mpro.

### 3.2 Representative drugs and their characteristics

N-0385. N-0385 is a small-molecule drug that has shown great promise in preclinical studies. Its unique chemical structure allows it to interact specifically with key proteins, TMPRSS2, involved in the SARS-CoV-2 infection process. In *in vitro* experiments using human lung cells, N0385 has been shown to significantly reduce the viral load. When added to cultures of donor-derived colon organoids infected with SARS-CoV-2, it effectively inhibits viral replication. What is particularly remarkable is its activity against different SARS-CoV-2 variants of concern (VOCs), such as B.1.1.7 and B.1.351 (21). These VOCs have mutations that make them more transmissible and potentially resistant to treatments. However, N-0385’s low-nanomolar potency means that it can inhibit the virus at very low concentrations, making it highly effective. Its high selectivity index indicates that it targets the virus with great precision, minimizing off-target effects on normal human cells. This combination of properties makes N-0385 a strong candidate for early-stage treatment of COVID-19, especially against emerging variants.

EDP-235. EDP-235 is a broad-spectrum coronavirus inhibitor that has been extensively studied in animal models. It targets proteins of SARS-COV-2 3CLpro involved in the assembly and release of new virus particles. In Syrian hamster models, which closely mimic the human respiratory tract infection, EDP-235 has been shown to reduce the viral load in the lungs significantly (22). In ferret models, EDP-235 not only inhibits viral replication but also reduces the severity of lung pathological changes. These changes include inflammation, tissue damage, and the presence of viral-induced lesions. The ability of EDP-235 to target both the primary infection and prevent virus transmission in these animal models makes it a promising candidate drug for further clinical development.

Nucleoside analog β-D-hydroxycytidine (NHC). β-D-hydroxycytidine (NHC) has attracted significant attention in the fight against SARS-CoV-2. Nucleoside analogs work by mimicking the natural nucleosides that the virus uses to replicate its RNA (23). When the virus incorporates NHC into its RNA chain during replication, it can cause errors in the genetic code or prematurely terminate the RNA synthesis process. NHC has shown strong antiviral activity against different coronaviruses, not just SARS-CoV-2. This broad-spectrum activity makes it a valuable candidate drug. By screening over 16,000 small interfering RNAs (siRNAs), super-effective siRNAs that can target the SARS-CoV-2 genome has been identified and these siRNAs, in combination with NHC, may provide a more potent antiviral strategy.

Nucleoside analog GS-441524, the active metabolite of remdesivir, binds active sites for nucleoside recognition and diphosphate binding of the RdRp and ExoN (19). By identifying the residues involved in the recognition process, more effective nucleoside analogs can be designed. These new analogs can be tailored to have a higher affinity for the active site, ensuring that they are preferentially incorporated into the growing RNA chain instead of the natural nucleosides. This research is important for emerging SARS-CoV-2 mutants and emerging coronaviruses.

### 3.3 New technology and targets

The PROTAC (proteolysis-targeting chimera) technology represents a revolutionary approach in small-molecule drug development. This technology takes advantage of the cell’s natural protein-degradation machinery, the proteasome. A PROTAC molecule consists of two functional domains: one that binds to a target protein (in this case, a viral protein) and another that binds to an E3 ubiquitin ligase, an enzyme involved in the ubiquitination process that marks proteins for degradation. The MDP2 compound, developed using PROTAC technology, has been shown to effectively reduce the M protein level in 293T cells (24). The M protein is essential for the assembly and budding of coronaviruses. By degrading the M protein, MDP2 disrupts the virus’s life cycle, preventing the formation of new infectious virus particles. This approach offers several advantages over traditional small-molecule inhibitors. For example, it can target proteins that are difficult to inhibit using traditional methods, and it may be less prone to drug resistance as it degrades the target protein rather than just inhibiting its activity.

New target. Identifying new drug targets is crucial for developing more effective antiviral drugs, especially in the face of emerging virus strains and species (25). The protein compound 17 is involved in the viral RNA capping process, essential for the stability and translation of viral RNA. By targeting this protein, drugs can potentially disrupt the virus’s ability to replicate and infect new cells. Combining drugs that target different stages of the viral life cycle, such as nirmatrelvir (25), targeting 3CLpro and other proteins, can reduce the risk of drug resistance.

Carrimycin, a recently approved macrolide antibiotic and a potential antiviral drug for SARS-CoV-2 in phase III studies, reduces the effectiveness of e1 ribosomal frameshifting. Carrimycin reduces the quantity of the essential elements of the viral replication and transcription complexes by directly binding to the coronaviral frameshift-stimulatory element (FSE) RNA pseudoknot, which stops the viral protein translation transition from ORF1a to ORF1b (26).

Aptamers are single-stranded DNA or RNA molecules, that have the ability to identify and attach to targets by folding into certain three-dimensional configurations and have shown great promise in the diagnosis and treatment of COVID-19 (27). In cell studies, RNA aptamers can effectively prevent infections by Delta and other variations by blocking viral spike proteins from attaching to ACE2 receptors (28). The chemical synthesis of aptamers also exhibits high stability. Besides, Aptamers can also be used in conjunction with nanotechnology and CRISPR. There are now extremely sensitive detection techniques, including the RBD-1CM1 aptamer, which has a far higher affinity for the S protein. Interestingly, this work used computer simulations to design and screen aptamers that target the SARS-CoV-2 spike protein (S protein). The RBD domain of the S protein was the only focus of the simulations, which ignored any possible conformational changes. Long-term dynamic interactions may not have been adequately captured by the simulations due to computing resource constraints (27, 29).

### 3.4 Challenges

The current treatment landscape for COVID-19 is hampered by a scarcity of approved small-molecule antiviral drugs. With only eight drugs available—four nucleoside analogs and four protease inhibitors—it’s difficult to manage the wide variety of coronaviruses.

Antiviral resistance poses a significant threat. As the virus replicates, its genome accumulates mutations that can reshape viral proteins, rendering existing drugs ineffective. For instance, mutations in the protease gene can modify the protease’s active site, blocking protease inhibitors from binding and halting the virus (20). This not only weakens treatment efficacy but also promotes viral spread.

Toxicity also limits the use of certain small-molecule antiviral drugs. They can cause harm to various organs; for example, long-term use of some nucleoside analogs can damage the kidneys. This restricts their long-term application, particularly for patients requiring continuous treatment. Some antivirals may interfere with liver enzymes involved in drug metabolism, either reducing the antiviral’s effectiveness if it’s metabolized too fast or increasing toxicity when its levels in the body spike.

Drug-drug interactions are another major concern. Many COVID-19 patients take medications for comorbidities like hypertension, diabetes, or heart disease. When used alongside small-molecule antivirals, interactions can occur.

To address these issues, ongoing research aims to combat drug resistance, to develop safer drugs, and gain a deeper understanding of drug-drug interactions.

## 4 Macro-molecule drugs against broad-spectrum of coronaviruses

### 4.1 Antiviral peptides and polymers

Antiviral peptides and polymers are an emerging class of antivirals with great potential. These macromolecules have diverse structures, which are determined by their amino acid or monomer sequences. Antiviral peptides, for example, can range from short chains of amino acids to long, complex polypeptides. Their structures can be linear, cyclic, or even branched, and they can adopt various secondary and tertiary structures, such as alpha-helices, beta-sheets, or random coils.

This structural diversity gives them a wide range of antiviral mechanisms. Antiviral peptides can prevent virus attachment to host cells via the spike protein, interfere with the fusion of the virus membrane with the host cell membrane, interfere with viral nucleic acid replication or inhibit viral protein synthesis.

Researchers are actively exploring ways to optimize these macromolecules for better antiviral activity. One approach is to modify their structures to enhance their stability and solubility. For example, adding chemical groups to the peptide or polymer backbone can improve its resistance to degradation by enzymes in the body and increase its ability to reach the site of infection. Another approach is to develop coatings or delivery systems that can protect these macromolecules and ensure their targeted delivery to infected cells.

### 4.2 Polysaccharide macromolecules in traditional Chinese medicine

Polysaccharides are complex carbohydrates composed of multiple sugar units linked together. Polysaccharide macromolecules derived from Traditional Chinese medicine (TCM) herbs have shown potential anti-coronavirus activity. For example, polysaccharides isolated from Astragalus membranaceus was found to have antiviral effects *in vitro* (30). However, the exact mechanisms by which these polysaccharide macromolecules exert their antiviral effects are not fully understood (31). They may activate immune cells, such as macrophages, T cells, and B cells (32, 33), or directly bind to the virus’s surface proteins or viral replication proteins.

### 4.3 Lipopolysaccharide-binding protein

Lipopolysaccharide-binding protein (LBP) plays an interesting role in viral infections. Through surface plasmon resonance (SPR), it has been discovered that LBP can bind to both the Spike protein of SARS-CoV-2 and the ACE2 protein on host cells, and disrupts the Spike-ACE2 interaction (34). Moreover, the binding of LBP can trigger membrane fusion in a way that is different from the normal viral entry process, making it a potential broad-spectrum virus entry inhibitor. In the context of respiratory virus infections, LBP may be developed into a nasal mucosa protectant. By applying LBP-based formulations in the nasal cavity, it may be possible to prevent the virus from infecting the respiratory epithelium. However, further research is needed to optimize the delivery and stability of LBP-based products and to fully understand its long-term effects on the human body.

### 4.4 Macromolecule CD-SA

CD-SA consists of a β-cyclodextrin scaffold modified with hydrophobic linkers in the primary face, onto which unitary sialic acid epitopes are covalently grafted to mimic influenza virus-host receptors. It can effectively inactivate these viruses, preventing them from infecting host cells by disrupting the virus’s envelope structure or interfering with its key proteins involved in the infection process. What is even more interesting is that CD-SA also shows antiviral activity against SARS-CoV-2. SARS-CoV-2 relies on sialic acid on the surface of host cells for attachment. CD-SA may interfere with this attachment process, either by binding to sialic acid receptors or by directly interacting with the Spike protein of SARS-CoV-2, preventing the virus from entering the cell (35).

## 5 Carrier vector vaccines against broad-spectrum of coronaviruses

### 5.1 Virus-like particles

Vaccines based on SARS-CoV-2 virus-like particles (VLPs) are at the forefront of vaccine development. VLPs are non-infectious particles that mimic the structure of the actual virus. They are composed of viral proteins, with the S protein being a key component. The S protein is responsible for the virus’s attachment to host cells, and it is also a major target for the immune system (36). When introduced into the body, VLPs are recognized as foreign invaders by antigen-presenting cells (APCs), mainly dendritic cells. These APCs take up the VLPs, process the S protein, and present it to T cells and B cells. This interaction activates the immune system, leading to the production of antibodies and the activation of T cells. The antibodies produced can bind to the S protein of the actual virus, preventing it from attaching to and infecting host cells. T cells, on the other hand, can recognize and kill virus-infected cells.

### 5.2 Adenovirus type 5

Adenovirus type 5 (Ad5) (37, 38) as a vector have shown great potential in combating SARS-CoV-2. Adenoviruses are well-studied vectors that have been used in vaccine development for many years. They have the ability to efficiently deliver foreign genes into host cells. In the case of Ad5-based SARS-CoV-2 vaccines, the gene encoding the viral S protein is inserted into the Ad5 genome.

The strategy of using intramuscular injection followed by intranasal booster vaccination is particularly effective. Intramuscular injection allows the vaccine to be distributed systemically, leading to the production of high levels of IgG antibodies. IgG antibodies are the most abundant antibodies in the blood and can circulate throughout the body, neutralizing the virus if it enters the bloodstream. Intranasal booster vaccination, on the other hand, targets the respiratory mucosa, which is the primary site of SARS-CoV-2 infection. This route of administration induces the production of IgA antibodies, which are important for mucosal immunity. IgA antibodies can prevent the virus from attaching to and infecting the respiratory epithelial cells. Additionally, this vaccination strategy also induces high levels of pseudovirus-neutralizing antibodies (PNAb) and Th1-biased T-cell responses. Th1-biased T-cell responses are important for cell-mediated immunity, which can help in clearing virus-infected cells.

### 5.3 Recombinant rabies virus

RABV has been engineered to express a tandem RBD heterotrimer as a multivalent immunogen (39). The RBD (receptor-binding domain) of the SARS-CoV-2 Spike protein binds to the ACE2 receptor on host cells.

### 5.4 Lumpy skin disease virus

LSDV is a poxvirus that has been modified to carry the stable Spike protein and conserved nucleocapsid protein of SARS-CoV-2 as antigens (40). The Spike protein is crucial for inducing neutralizing antibodies, while the nucleocapsid protein can stimulate a cell-mediated immune response. The use of LSDV as a carrier has the advantage of its ability to elicit a strong immune response, and it may also have potential for long-term immunity due to the nature of poxvirus-based vaccines.

### 5.5 Recombinant parainfluenza virus

rVSV has shown good immune effects in pre-clinical studies. rVSV is a well-studied virus that has been engineered to express the SARS-CoV-2 Spike protein. Intranasal vaccination with this vaccine is an attractive approach as it directly targets the respiratory mucosa, the primary site of SARS-CoV-2 infection (41).

When the rVSV-based vaccine is administered intranasally, it induced the production of IgA and IgG antibodies and the activation of T cells. The good immune effects observed in mice and non-human primates suggest that this vaccine has potential for human use, and further clinical trials are underway to evaluate its safety and efficacy.

### 5.6 SARS-CoV-2 delta strain

The Delta strain of SARS-CoV-2 is a highly transmissible variant that has posed new challenges to vaccine development. However, researchers are leveraging this strain to develop more effective vaccine carriers. By optimizing the carrier, codons, and protein structure, they aim to create vaccines that can provide broad-spectrum protection against not only the Delta strain but also other variants.

Optimizing the carrier involves modifying the vector to improve its stability, delivery efficiency, and immunogenicity. Codon optimization is used to enhance the expression of the Spike protein or other antigens in the host cells. Protein structure optimization focuses on making the antigen more recognizable by the immune system. For example, mutations in the Spike protein of the Delta strain can be incorporated into the vaccine antigen in a way that enhances its immunogenicity. These efforts are expected to lead to the development of vaccines that can effectively protect against the evolving SARS-CoV-2 variants.

### 5.7 Antiviral effects of engineered cells

Researchers have made significant progress in developing engineered cells that can produce broad-spectrum antiviral effects. These engineered cells are created through genetic modification techniques, such as gene editing and transfection. By introducing specific genes into the cells, they are endowed with the ability to recognize and inhibit viruses (42).

One common approach is to engineer cells to express antiviral proteins. For example, cells can be engineered to express interferons, which are natural proteins produced by the body in response to viral infections. Interferons have broad-spectrum antiviral activity. These responses include the activation of genes that inhibit viral replication, the upregulation of immune-related molecules, and the enhancement of the immune system’s ability to recognize and clear virus-infected cells.

Another approach is to engineer cells to express receptors or antibodies on their surface that can specifically bind to viruses. These receptors or antibodies can act as decoys, preventing the virus from binding to and infecting normal host cells. For example, cells can be engineered to express the ACE2 receptor.

These engineered cells provide a new means for pandemic preparedness. In the event of a viral outbreak, these cells could potentially be used in various ways. They could be used to produce antiviral proteins or antibodies on a large scale for therapeutic use. They could also be used in cell-based therapies, where the engineered cells are directly administered to patients to enhance their antiviral defenses. Although these engineered cells are still in the experimental stage, their potential application value in future antiviral treatments is significant, and further research is being conducted to optimize their antiviral effects and safety.

### 5.8 Significance of carrier-based vaccines

The research and development of carrier-based vaccines are of utmost importance in the fight against coronaviruses. The virus’s rapid mutation and widespread transmission have made it a formidable opponent. Carrier-based vaccines offer a promising solution to these challenges.

Carrier-based vaccines also aim to provide more durable immune effects. Some carriers, such as adenoviruses and poxviruses, have the ability to persist in the body for a certain period, continuously presenting antigens to the immune system. This can lead to the development of long-term immunity, reducing the need for frequent booster vaccinations.

Reducing virus transmission is a key goal of carrier-based vaccines. When a large proportion of the population is vaccinated with carrier-based vaccines, it can create herd immunity. Herd immunity occurs when a sufficient number of people are immune to the virus, making it difficult for the virus to spread from person to person. This can effectively protect those who are not vaccinated, such as individuals with weakened immune systems or those who cannot receive vaccines for medical reasons.

In the context of global epidemic prevention and control, carrier-based vaccines have important strategic significance. They can be produced on a large scale and distributed globally, helping to control the spread of the virus across different regions.

## 6 Traditional Chinese medicine against broad-spectrum of coronaviruses

### 6.1 Overview

TCM has a long-standing history of treating infectious diseases. By synthesizing traditional medical literature, modern studies, and real-world clinical experiences, “TCM Protocol” for COVID-19 was established (43). This protocol was not a one-size-fits-all approach but rather a comprehensive set of guidelines that took into account the different stages of the disease, the patient’s constitution, and the overall clinical manifestations.

Clinical statistics have provided compelling evidence of the effectiveness of TCM in the treatment of COVID-19. In China, a large number of COVID-19 patients received integrated traditional Chinese and Western medicine treatment. The data showed that these patients had lower mortality rates compared to those treated with modern medicine alone (44). Moreover, they also experienced relatively better clinical outcomes, such as faster recovery times, reduced severity of symptoms, and fewer complications. For instance, many patients reported alleviation of symptoms like fever, cough, and fatigue more rapidly when TCM was incorporated into their treatment regime. This not only demonstrated the efficacy of TCM in treating COVID-19 but also highlighted the potential of integrating traditional and modern medicine to combat complex diseases.

### 6.2 Representative TCM

#### 6.2.1 Glycyrrhizic acid (ZZY-44)

Glycyrrhizic acid (ZZY-44), a compound derived from the root of Glycyrrhiza species, has emerged as a promising broad-spectrum anti-coronavirus molecule. *In vitro* studies have shown that it exhibits significant inhibitory effects against SARS-CoV-2 (45). The unique chemical structure of glycyrrhizic acid allows it to interact with various components of the virus and the host cell. It interferes with the virus’s ability to attach to host cells by binding to the Spike protein of SARS-CoV-2, preventing the initial step of infection. Additionally, glycyrrhizic acid has immunomodulatory properties. It can enhance the body’s immune response by activating immune cells, such as macrophages and lymphocytes, which play a crucial role in the fight against viral infections. These dual-acting mechanisms make glycyrrhizic acid a potential candidate for further development as an antiviral agent, either as a stand alone treatment or in combination with other drugs.

#### 6.2.2 Lianhua Qingwen Granules (LHQK)

Lianhua Qingwen Granules (LHQK), which has been approved for treating acute tracheobronchitis, has also shown remarkable broad-spectrum antiviral effects against COVID-19 (46). This TCM formulation contains a blend of multiple herbs, including forsythia, honeysuckle, ephedra, and almonds, among others. The combination of these herbs works synergistically to inhibit viruses. Research has indicated that LHQK can directly target the virus, interfering with its replication process at the molecular level. It also has the ability to modulate the host’s anti-inflammatory responses. In animal models and clinical trials, LHQK treatment led to a reduction in weight loss and lung index, which are indicators of disease severity. By suppressing excessive inflammation in the lungs, LHQK helps to protect the lung tissue from damage caused by the virus and the body’s own immune response.

Another study also showed that Lianhua Qingwen (LHQW) polarizes macrophages toward anti-inflammatory phenotypes while promoting M2 macrophage recruitment in LPS-induced acute lung injury (ALI) *in vitro*. This dual modulation offers new insight on how LHQW simultaneously resolves inflammation and eliminates viruses to protect against ALI (47). This dual-effect mechanism not only combats the virus but also alleviates the symptoms associated with COVID-19, making it a widely used and effective treatment option.

#### 6.2.3 PGG and TGG from Terminalia chebula

PGG (pentagalloylglucose) and TGG (tannic acid-related gallotannin) are compounds identified from Terminalia chebula, a plant with a long history of use in traditional medicine. These two compounds have shown great promise as dual-target inhibitors of SARS-CoV-2 (48). PGG and TGG can simultaneously target multiple key proteins or pathways in the virus’s life cycle. This dual-targeting strategy is particularly significant as it may help to avoid drug resistance. When a drug targets only one aspect of the virus, the virus can relatively easily develop mutations to evade its effects. However, by targeting two different aspects, the virus has a much harder time developing resistance. In pre-clinical studies, PGG and TGG have demonstrated strong inhibitory effects on SARS-CoV-2 replication. Their unique mechanism of action and potential to overcome drug resistance make them exciting candidates for future anti-COVID-19 drug development.

#### 6.2.4 FZJDD (a compound TCM)

FZJDD, a complex traditional Chinese medicine formulation, has broad- spectrum anti-coronavirus activity (49). It is composed of multiple herbs, each contributing to its overall antiviral effect. The exact mechanisms and material basis of FZJDD’s anti-COVID-19 activity are gradually being elucidated through extensive research. Scientists are using advanced analytical techniques to identify the active components within FZJDD and understand how they interact with the virus and the host. FZJDD may act through a combination of direct antiviral effects, such as inhibiting viral entry and replication, and immunomodulatory effects, enhancing the body’s ability to fight the infection. As the research progresses, a more comprehensive understanding of FZJDD will enable further optimization of its formulation and application in the treatment of COVID-19.

Turmeric root has long been used in natural therapy. Curcumin, its bioactive component, has broad-spectrum antimicrobial action. Researchers discovered that SARS-CoV-2 was successfully neutralized at sub-toxic quantities in Vero E6 and human Calu3 cells by turmeric root extract, dissolved turmeric capsule contents, and pure curcumin. Additionally, in cell culture, curcumin dramatically decreased SARS-CoV-2 RNA levels. Curcumin shows promise as a supplemental COVID-19 treatment, according to the study’s findings (50). Curcumin exhibits antiviral activity against multiple viruses, including influenza A virus, HIV, HCV and SARS-CoV-1 (51–53). Curcumin demonstrates both anti-inflammatory and antiviral properties. Its supplementation has been shown in a randomized controlled trail to dramatically reduce human interleukin-6 (IL-6) and tumor necrosis factor-alpha (TNFα) (54, 55). However, Curcumin can only be absorbed by the humans body at a rate of 1%, followed by a half-life of around 8 hours, it is broken down into a variety of inactive metabolites. Therefore, curcumin’s therapeutic use may be restricted by its limited bioavailability (50, 56, 57).

The Omicron RBD has 15 important mutations that increase the virus’s affinity for ACE2, including S375F, S477N, S371L, S373P, N440K, N501Y, K417N, T478K, G339D, G496S, G446S, E484A, Q498R, Q493R, and Y505H (58, 59). By attaching to certain mutation sites (such as Ser496, His505, and Arg493), flavonoids can disrupt the crucial interface of the RBD-ACE2 interaction. Flavonoids may prevent viral multiplication within host cells, restrict viral attachment and endocytosis, and lessen the chance of immunological escape by blocking RBD-ACE2 binding (by locking the RBD conformation, hence preventing mutations in antibody recognition sites). Flavonoids inhibit the function of the Omicron RBD through a triple mechanism involving multi-target binding, conformational stabilization, and high affinity. Among these, compounds such as Tomentin A and Corylifol A emerge as potential drug candidates due to their strong binding energies (-8.7 to -8.2 kcal/mol) and low toxicity (LD50 1000–5000 mg/kg). However, their antiviral activity and safety require further validation through *in vitro* experiments. The core mechanism by which flavonoids inhibit the Omicron variant involves binding to key mutation sites on the RBD of the spike protein, thereby blocking viral interaction with the host cell surface ACE2 receptor and inhibiting viral entry (60). The RBD active site is the main target of current medications, however viral mutations often cause treatment resistance, requiring the creation of new allosteric inhibitors to maintain the RBD in its “down” conformation. A conserved allosteric region in the spike protein has been shown to be a possible broad-spectrum antiviral target. Researchers have identified CPD3, CPD5, and CPD6 as allosteric modulators that prevent viruses from binding to ACE2 by stabilizing the RBD in its “down” conformation. This offers a novel strategy to the development of anti-SARS-CoV-2 medications, especially those that may be broad-spectrum effective against variations. By avoiding the resistance problems with conventional RBD-targeting medications, allosteric inhibitors provide fresh perspectives on COVID-19 therapy. However, this work only uses computational validation; compound activity and *in vivo* efficacy must be confirmed experimentally. Allosteric inhibitors provide new perspectives for COVID-19 therapy by avoiding the resistance problems linked to conventional RBD-targeting medications. Nevertheless, experimental proof of chemical activity and *in vivo* effectiveness is necessary for this work, which only uses calculation. In order to improve affinity, compound structures may be further optimized, and combination treatment approaches may be investigated (61).

Mpro is an important enzyme that is essential for viral transcription and replication, which makes it a possible target for treatment of SARS-CoV-2 infection (62). Through computational methods, researchers identified effective inhibitors of the SARS-CoV-2 main protease. Molecular docking technology was employed to test compounds from a phytochemical library against the main protease, screening for the most active lead compounds. Furthermore, the top three ligands from the phytochemical library—cosmosiine, pelargonidin-3-O-glucoside, and cleomiscosin A—were found to bind to the active site of the target protein. The three ligand molecules (Cosmosiine, Pelargonidin-3-O-glucoside, Cleomiscosin A) achieve binding by forming hydrogen bonds and hydrophobic interactions with key residues in the active site of the SARS-CoV-2 main protease (Mpro). All three ligands bind to the catalytic pocket of Mpro (composed of Cys145 and His41), inhibiting enzyme activity by blocking substrate binding. By precisely targeting the active site and exhibiting multi-type interactions, these three plant compounds synergistically function as potential Mpro inhibitors, providing a structural foundation for anti-COVID-19 drug development. As this study relies solely on computational mining, these findings require further validation through computational cross-docking and *in vitro* experiments, including enzyme activity assays (63).

Jadwar is a significant medicinal plant in traditional Persian medicine, rich in diterpene alkaloids. Researchers employed molecular docking and molecular dynamics simulation techniques to analyze the binding affinity of alkaloids derived from Jadwar with SARS-CoV-2 Mpro. This study represents the first systematic evaluation of the anti-COVID-19 activity of alkaloids from the Jadwar plant, offering new insights for natural product drug development. However, this study did not account for solvent effects or the influence of protein flexibility on binding modes. *In vitro* antiviral activity and cytotoxicity of the compounds are required to be validated through wet-lab experiments (64).

In order to find possible Mpro inhibitors, researchers used molecular modeling techniques to screen natural plant chemicals, offering a theoretical basis for COVID-19 drug development. They found that oleanolic acid, apigenin, and ursolic acid bind to Mpro via hydrophobic interactions and stable hydrogen bonds; ursolic acid has the highest binding energy (-168.92 kJ/mol) and, thus, the most potential as an inhibitor. This justifies further *in vitro/in vivo* validation and offers theoretical basis for the development of anti-COVID-19 medications derived from plants. Nevertheless, the work ignores the effect of Mpro mutations in viral variations and only uses computational simulations without experimental validation (65).

### 6.3 Mechanisms

The therapeutic mechanisms of TCM in treating COVID-19 are characterized by multi-target and holistic regulation, which sets it apart from conventional modern medicine. On one hand, TCM has the ability to regulate the human immune system. Many TCM herbs and compounds can enhance the body’s resistance to viral infections. For example, some herbs can activate macrophages, which are the body’s first line of defense against pathogens. Activated macrophages can engulf and digest viruses, as well as secrete cytokines that further activate other immune cells. TCM can also modulate the function of T-lymphocytes and B-lymphocytes.

On the other hand, the active components in TCM may directly act on the virus. Some TCM compounds can inhibit viral replication by interfering with the virus’s enzymes or proteins involved in the replication process. For example, certain alkaloids and flavonoids can bind to the viral RNA-dependent RNA polymerase, which is essential for the virus to replicate its genetic material. By binding to this enzyme, these compounds can prevent the virus from making copies of itself. TCM components can also inhibit viral invasion. They may interfere with the interaction between the virus’s Spike protein and the host cell’s ACE2 receptor, preventing the virus from entering the cell.

### 6.4 Modern research methods in TCM

In the quest to unlock the full potential of TCM, modern technologies such as high-performance liquid chromatography (HPLC) and high-resolution mass spectrometry (HRMS) have been instrumental. For example, research on QFPD found that leupeptin decoction contributes to the effective treatment of COVID-19 (66). Leupeptin is a protease inhibitor that may play a role in inhibiting the viral proteases involved in the replication process of SARS-CoV-2. By using HPLC and HRMS, researchers were able to identify leupeptin in the QFPD decoction and study its concentration and potential mechanism of action.

These technological methods are crucial for clarifying the chemical components and mechanisms of TCM. They help researchers to understand which components in a TCM formulation are responsible for its antiviral and immunomodulatory effects. This knowledge is essential for standardizing the production of TCM drugs, ensuring their quality and efficacy. By identifying the active components, researchers can also develop more targeted TCM treatments and explore new ways to enhance their effectiveness. In the context of anti-COVID-19 research, these technologies are promoting the modernization of TCM, bridging the gap between traditional medical wisdom and modern understanding, and providing a more basis for the application of TCM in the fight against coronaviruses. We summarizes recently published studies on the effects and mechanisms of TCM in the treatment of coronaviruses in **Table 1**.

**Table 1:** Evidence and mechanisms of the traditional Chinese medicine.

Category	Active ingredient	Methods and subject	Role or function	Mechanism	Ref
*Salvia* *miltiorrhiza*	Danshensu	Mouse model	In a mouse model, danshensu reduced lung inflammation, and among lipopolysaccharide (LPS)-stimulated RAW264.7 macrophages, danshensu derivatives demonstrated superior anti-inflammatory action.	The catechol ring of Danshensu is the site of the covalent interaction with SARS-CoV-2 3CLpro.	(97)
Andrographis paniculta and scutallaria baicalensis	Andrographolide and baicalin	Mice model	Andrographolide-baicalin combination targets multiple COVID-19 entry points: reducing ACE2 availability, increasing spike-ACE2 binding avidity, inhibiting viral attachment kinetics, and suppressing proinflammatory cytokine release (IL-6, TNF-α).	Andrographolide and baicalin exhibit cooperative suppression of coronavirus replication pathways through attenuation of ACE2 expression levels.	(98)
Xuebijing injection, Tanreqing capsule, lingmao formula, and Baqi Lingmao formula	Methyl rosmarinate	Laboratory testing, virtual screening, and experimental substantiation.	Methyl rosmarinate interacts with the allosteric binding pocket of SARS-CoV-2 3CL protease.	Methyl rosmarinate functions as an allosteric modulator of SARS-CoV-2 3CL protease, effectively blocking viral replication through interaction with the replicon system.	(99)
Gan Cao	Glycyrrhetinic acid (GA)	*In vitro* and mice modlle.	Inflammation-suppressive effects, oxidative stress mitigation, viral replication inhibition.	GA exerts its virucidal effects via direct engagement of the STING protein, thereby stimulating the cGAS-STING signaling axis.	(100)
Natural plants and Chinese herb medicine	myricetin	LPS-induced acute lung injury (ALI) animal models	Myricetin Disrupts Spike-ACE2 interactions, inhibiting viral entry.	Myricetin exhibits high-affinity binding to SARS-CoV-2RBD^WT^, disrupting ligand-receptor engagement with ACE2.	(16)
Xuanfei Baidu Decoction (XFBD)		LPS-induced ALI mouse model	XFBD administration alleviates pulmonary consolidation and edema in LPS-induced ALI mice through anti-inflammatory effects, manifested by significant reductions in proinflammatory cytokines IL-6 and TNF-α.	XFBD disrupts neutrophil trafficking in LPS-stimulated ALI via dual mechanisms: CXCL2/CXCR2 chemokine signaling inhibition and migratory pathway suppression, leading to NETosis attenuation and PNA synthesis reduction.	(101)
Lianhua Qingwen (LHQW)		LPS-induced ALI mouse model	LHQW simultaneously resolves inflammation and eliminates viruses to protect against ALI.	LHQW polarizes macrophages toward anti-inflammatory phenotypes while promoting M2 macrophage recruitment.	(47)

## 7 Comparative analysis of various drug categories

### 7.1 Comparative analysis

**Figure 1:**
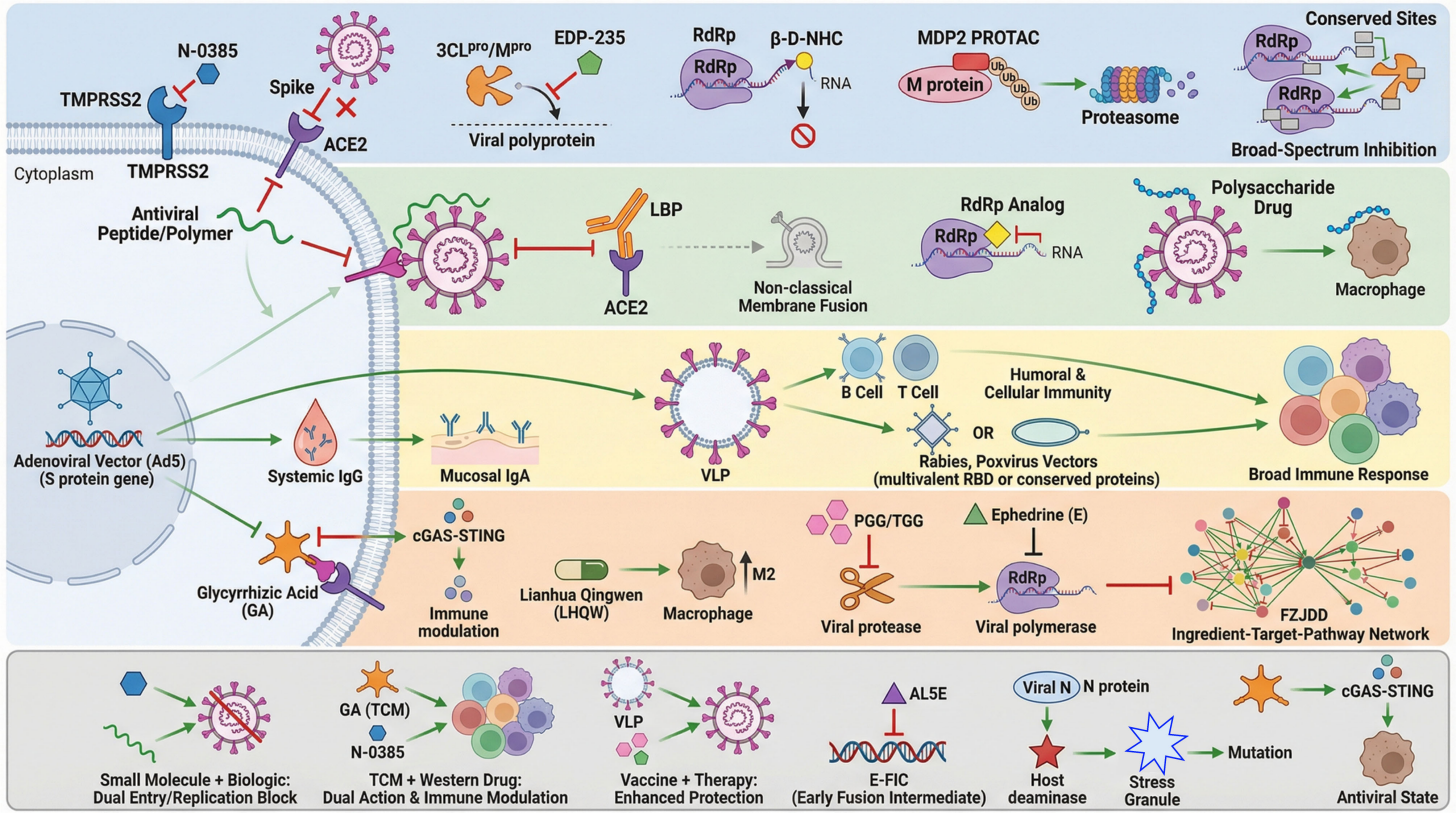
Schematic illustration of the integrated antiviral and immunomodulatory mechanisms of Traditional Chinese Medicine (TCM) compared with modern therapeutics against SARS-CoV-2. The mechanism diagram was generated by Gemini 3. The authors provided relevant information as prompts. The initial draft generated by the AI was refined using Adobe Illustrator for details and biological accuracy verification, ensuring consistency with experimental data. NHC, Nucleoside analog β-D-hydroxycytidine (NHC); PROTAC, proteolysis-targeting chimera; RdRp, RNA-dependent RNA polymerase; TCM, Traditional Chinese Medicine; LBP, Lipopolysaccharide-binding protein; FZJDD, a compound TCM; PGG, pentagalloylglucose; TGG, tannic acid-related gallotannin; VLP, Virus-like particle; E-FIC, early fusion intermediate conformation; AL5E, E-FIC-targeted bifunctional antiviral protein.

**Figure 1** showed the broad-spectrum anti-SARS-Cov-2 mechanisms. **Table 2** showed comparison among four major classes of therapeutics against coronaviruses. Small-molecule drugs offer precise targeting of viral proteins with easily modifiable structures. The macromolecular drugs provide multi-stage viral inhibition through complex peptide/polymer structures. traditional Chinese medicine delivers holistic effects through multi-component formulations that address both viruses and host immunity. While vector vaccines generate comprehensive and durable protection by inducing both antibody and T-cell responses.

**Table 2:** Comparison of anti-coronaviruses drug classes of small molecules, macromolecules, traditional Chinese medicine and vector vaccines.

Drug type	Characteristics	Advantages	Example/Applications
Small-Molecule Drugs	Well-defined structures, clear mechanisms of action, easy synthesis and modification.	Precise targeting of viral proteins (e.g., Mpro, RdRp); tunable properties.	N-0385 (inhibits variants in lung cells & organoids), EDP-235 (broad-spectrum, reduces lung damage).
Macromolecular Drugs	Diverse structures (peptides, polymers), multi-stage viral targeting.	Blocks viral entry/replication; lowers resistance risk via multi-site binding.	Antiviral peptides (disrupt envelopes/nucleic acids), Polymers (trap viruses).
Traditional Chinese Medicine (TCM)	Multi-component (polysaccharides, glycyrrhizic acid), effectiveness.	Immune modulation, symptom relief, direct antiviral action.	Glycyrrhizic acid (anti-coronavirus), reduces fever/cough/fatigue, enhances recovery.
Vector Vaccines	Use viral vectors (e.g., adenovirus) to deliver antigens.	Stimulates both humoral & cell-mediated immunity; long-lasting protection.	Adenovirus-based vaccines (induce IgG/IgA, Th1-biased T-cells, pseudovirus neutralization).

Small molecules excel in specificity and tunability, macromolecules in multi-target action, TCM in multi-functional benefits, and vaccines in preventive efficacy. Representative examples include N-0385 (small-molecule inhibitor), glycyrrhizic acid (macro-molecule inhibitor), adenovirus-based vaccines and LHQW (TCM). The approaches differ fundamentally in their purposes (treatment *vs* prevention) and mechanistic complexity, ranging from single-target drugs to systemic immune activators. Further considerations might include administration routes and clinical development stages.

### 7.2 Exploration of combination therapies

Combination therapies show promise against COVID-19 by leveraging complementary mechanisms of different drug classes. Small-molecule drugs (targeting viral enzymes) combined with macromolecular drugs (blocking entry/immune modulation) create synergistic effects-rapid viral suppression coupled with broader antiviral action. For example, antiviral peptides may enhance immune responses while polymers prevent cellular attachment. Integrating TCM with modern drugs offers dual benefits: reducing drug toxicity (e.g., hepatotoxicity) through metabolic regulation while enhancing antiviral efficacy via TCM’s immunomodulatory components. This approach may improve treatment tolerability and effectiveness. Vaccine-drug combinations provide comprehensive protection: vector vaccines prevent infection through immune priming, while therapeutic drugs manage breakthrough cases. This dual strategy addresses both prevention and treatment needs.

Further clinical studies are needed to establish optimal combination protocols balancing safety and therapeutic outcomes.

## 8 AI−based screening and computational strategies for broad−spectrum anti−coronavirus drug discovery

The rapid emergence of SARS−CoV−2 variants and the continuous threat posed by existing and emerging coronaviruses highlight the urgent need for efficient strategies to identify broad−spectrum antiviral agents. In recent years, artificial intelligence (AI)–based and computational approaches have become powerful tools for accelerating antiviral drug discovery by enabling large−scale screening, target prioritization, and rational optimization of candidate molecules (67–69). These approaches are particularly valuable for both small chemical molecules and macromolecular therapeutics, where experimental screening alone is often time−consuming and resource−intensive.

### 8.1 AI−assisted screening of small−molecule inhibitors

AI−driven virtual screening has been widely applied to identify small−molecule inhibitors targeting key conserved coronavirus proteins, including the main protease (Mpro/3CLpro) (70, 71), RNA−dependent RNA polymerase (RdRp) (72, 73), papain−like protease (PLpro) (74, 75), and the Spike protein (76, 77). By integrating molecular docking, molecular dynamics simulations, and machine−learning–based scoring functions, researchers can rapidly evaluate thousands to millions of compounds from synthetic, natural product, or repurposed drug libraries.

Recent studies have demonstrated that AI−guided screening can efficiently identify potential broad−spectrum inhibitors with favorable binding profiles against multiple coronavirus variants (69, 78, 79). Structure−based deep learning models and *de novo* drug design algorithms have further enabled the generation of novel chemical scaffolds optimized for binding affinity, selectivity, and physicochemical properties (80, 81). In addition, AI−assisted approaches have facilitated the discovery of allosteric inhibitors that stabilize viral proteins in inactive conformations, providing alternative strategies to overcome resistance associated with mutations at catalytic or receptor−binding sites (82, 83). Despite these advances, most AI−identified small−molecule candidates remain at the computational or early experimental validation stage. Therefore, integration of AI predictions with biochemical assays, cell−based antiviral assays, and *in vivo* studies is essential to confirm antiviral efficacy and safety.

### 8.2 Machine learning–guided discovery of natural products and phytochemicals

Natural products and traditional medicinal compounds represent a rich source of chemically diverse antiviral candidates. AI−based methods, including machine learning classifiers, network pharmacology, and multi−target prediction models, have been increasingly employed to screen phytochemical libraries for anti−coronavirus activity (84, 85). These approaches are particularly suitable for identifying compounds with multi−target potential, which is advantageous for broad−spectrum antiviral activity.

Computational studies have successfully predicted flavonoids, alkaloids, terpenoids, and phenolic compounds capable of interfering with Spike–ACE2 interactions or inhibiting viral proteases such as Mpro (86–88). AI−assisted network analyses have further helped to elucidate potential host–virus interaction pathways modulated by these compounds, providing mechanistic insights into their antiviral and immunomodulatory effects. However, similar to small−molecule screening, many findings from in silico analyses require experimental confirmation to validate biological relevance (89, 90).

### 8.3 Computational design and optimization of macromolecules

AI−based and computational strategies are also increasingly applied to the discovery and optimization of macromolecular antivirals, including antiviral peptides, polymers, and nucleic acid aptamers. Machine learning models can predict peptide sequences with enhanced antiviral potency, stability, and reduced cytotoxicity by learning from known antiviral peptide datasets. These peptides may act by disrupting viral membranes, blocking Spike−mediated entry, or interfering with intracellular replication processes.

For nucleic acid aptamers, in silico selection and structural modeling approaches have enabled the rational design of aptamers targeting conserved regions of the SARS−CoV−2 Spike protein, particularly the receptor−binding domain (RBD) (91, 92). Computational simulations facilitate the evaluation of binding affinity and specificity, as well as the assessment of potential conformational changes. Although such AI−designed macromolecules show promise, their antiviral activity, pharmacokinetics, and immunogenicity must be validated through experimental studies.

### 8.4 Advantages, limitations, and future perspectives

AI−based screening offers several advantages in anti−coronavirus drug discovery, including high throughput, reduced cost, and the ability to rapidly adapt to newly emerging viral variants. By enabling the prioritization of candidates with broad−spectrum potential, AI approaches can significantly shorten the early stages of drug development (93, 94). However, limitations remain. AI models are highly dependent on the quality and diversity of training datasets, and computational predictions may not fully capture complex biological processes such as protein dynamics, host responses, or *in vivo* pharmacology (95, 96). Moreover, discrepancies between predicted binding affinity and actual antiviral efficacy are common.

## 9 Conclusions and future perspectives

The fight against coronaviruses has made significant progress in the development of broad-spectrum therapeutics, including small-molecule drugs, macromolecular drugs, vector vaccines, and traditional Chinese medicine (TCM). However, each approach faces distinct challenges that must be addressed to improve efficacy and adaptability.

Small-molecule drugs offer rapid deployment and scalability but are hindered by drug resistance, toxicity, and potential interactions with other medications. The high mutability of coronaviruses accelerates the emergence of resistant strains and species, limiting long-term utility. Macromolecular drugs (e.g., monoclonal antibodies) show promise but require further mechanistic elucidation. Their complex structures pose production and stability challenges, and their high specificity may be undermined by viral evolution. Vector vaccines remain critical for prevention but demand continuous updates to match emerging viral variants and species. Antigenic drift can reduce immune recognition, necessitating platform improvements or multivalent designs to sustain broad protection. TCM has demonstrated utility in clinical management, yet its mechanisms—particularly its multi-target, holistic effects—lack rigorous molecular characterization. Standardization of quality and composition across formulations is another concern.

It’s required to prioritize basic research to clarify drug-virus interactions, enable smarter drug design with enhanced efficacy and reduced side effects. For TCM, modern analytical techniques (e.g., metabolomics, network pharmacology) could decode its polypharmacology. Synergistic regimens (e.g., small molecules + macromolecules) may broaden antiviral coverage and curb resistance. Rigorous clinical trials are needed to identify optimal combinations. AI-driven drug discovery may accelerate virtual screening of compounds and predict mutational impacts. Big data analytics allow to mine real-world clinical datasets to optimize dosing and track variant-specific treatment outcomes. Invest in next-generation platforms (e.g., mRNA, nanoparticle-based) may enable rapid antigen updates and universal coronavirus protection. Strengthen partnerships for surveillance, data sharing, and equitable therapeutic distribution are required to combat disparities in pandemic responses.

In addition, AI−based screening and computational drug discovery are emerging as critical enablers for the rapid identification and optimization of both small−molecule and macromolecular broad−spectrum anti−coronavirus candidates, particularly by accelerating virtual screening, predicting mutational impacts, and guiding rational drug design in response to viral evolution.
